# Definition of a measurement technique for hexapod circular smart fixators' perioperative assembly parameters and investigation of alignment and correlation with postoperative measurements: a retrospective cohort study

**DOI:** 10.1186/s12891-024-08056-y

**Published:** 2024-11-20

**Authors:** Muharrem Kanar, Yusuf Sülek, Tolga Hayrettin Seymenoğlu, Raffi Armağan

**Affiliations:** Department of Orthopaedics and Traumatology, University of Health Sciences Şişli Hamidiye Etfal Education and Research Hospital, Istanbul, Turkey

**Keywords:** Taylor spatial frame, Hexapod, Computer assisted, Mounting parameter, Deformity correction, Intraoperative measurement, Postoperative measurement

## Abstract

**Background:**

With the assistance of smart fixator technologies, the correction of complex deformities has been facilitated; however, the accurate integration of specialized radiographs and measurements into the system remains the greatest disadvantage, necessitating specialized imaging and an experienced team. When inexperienced technicians and doctors perform these specialized postoperative radiographs, excessive exposure of the patient and team to radioactive rays exacerbates inadequacies in measurements and delays the correction of residual deformities due to angular and translational adjustments. In this study, we compared postoperative measurements with those taken peroperatively via fluoroscopy, hypothesizing that it reduces the exposure of the patient and team to radioactive rays, allows for more accurate and timely correction of deformities and assembly parameters, and reduces time and costs.

**Methods:**

Between 2013 and 2022, 84 patients with bone deformities were retrospectively reviewed. All patients had bone deformities and were treated with computer-assisted circular external fixator systems (Ca-CEF). Assembly parameter measurements began to be corrected via artificial neural network software via peroperative fluoroscopy in 37 patients and postoperative radiography in 47 patients. The surgical duration for all patients, peroperative measurement values, and number of radiographs taken on postoperative day 1, week, and month until deformity correction were recorded.

**Results:**

The duration until deformity correction was shorter in patients who underwent postoperative measurements (mean 50.24 days) than in those who underwent peroperative measurements (mean 42.31 days), but this difference was not statistically significant (*p* = 0.102). The surgical duration was significantly shorter in patients with postoperative measurements (mean of 130.37 min) than in those with peroperative measurements (mean of 155.88 min) (*p* = 0.045).

For patients with postoperative measurements, 56.04 postoperative radiographs were taken. In contrast, patients with peroperative measurements had fewer radiographs totaling 28.7. This difference was statistically significant (*p* < 0.01). There was no statistically significant difference in the fluoroscopy dose between patients with postoperative measurements (mean 18.54 mGy) and those with peroperative measurements (mean 22.22 mGy) (*p* = 0.105).

**Conclusion:**

To achieve accurate assembly parameters, minimizing X-ray exposure is crucial but can pose challenges. Our results showed that despite an average increase of 25 min in surgical duration, the time taken for deformity correction was shorter. Additionally, we obtained fewer postoperative radiographs, indicating reduced radiation exposure.

## Introduction

Circular external fixators have been used for many years to correct complex deformities. Over the past two decades, various computer-assisted circular external fixator systems (Ca-CEF) have been introduced [1]. These systems are utilized to treat fractures, nonunions, correct limb shortening, and deformities [2–4]. The mathematical basis of Ca-CEF systems lies in projective geometry, which defines the repositioning of an object in space in a complex manner [5]. The Taylor brothers developed one of the first six-axis correction systems, known today as the Taylor spatial frame (TSF; Smith & Nephew, Memphis, TN) [5, 6]. Various Ca-CEF systems based on similar principles have subsequently been adopted and used clinically. These systems represent significant advancements in orthopedic surgery, offering precise control over the correction of deformities and providing surgeons with tools to achieve accurate and reproducible results in complex cases.

Ca-CEF consists of 2 rings connected by 6 telescopic rods, allowing simultaneous deformity correction around a virtual hinge axis through computer-assisted six-axis deformity analysis [3–7]. It facilitates adjustment of the lengths of the six supports around a virtual hinge to correct a fracture or osteotomy site in all six axes simultaneously [3–7]. Properly calculating the deformity, frame, and assembly parameters and entering the correct parameters into the system are crucial for the perfect placement of this virtual hinge.

The analysis of deformity and assembly parameters is possible through postoperative, specially taken radiographs, computed tomography (CT), and measurements under peroperative fluoroscopy [8–10]. The greatest drawback of these options lies in the accurate transfer of these measurements to the system, which requires specialized imaging with an experienced team. When inexperienced technicians or doctors take these radiographs or when CT scans are performed, excessive exposure of radiation is inevitable [9]. Additionally, inadequate measurements can result in residual deformity due to remaining angular or translational discrepancies [3, 7, 9]. These disadvantages increase costs.

We hypothesize that measurements of deformity and assembly parameters performed peroperatively using fluoroscopy reduce radiation exposure for the patient and surgical team, provide more accurate measurements of deformity and assembly parameters, and reduce time and costs. We aimed to compare the surgery times, fluoroscopy doses, deformity correction times, and postoperative radiographs by comparing the measurements of mounting parameters made using fluoroscopy peroperatively with the measurements of postoperative mounting parameters.

## Materials and methods

**Fig. 1:**
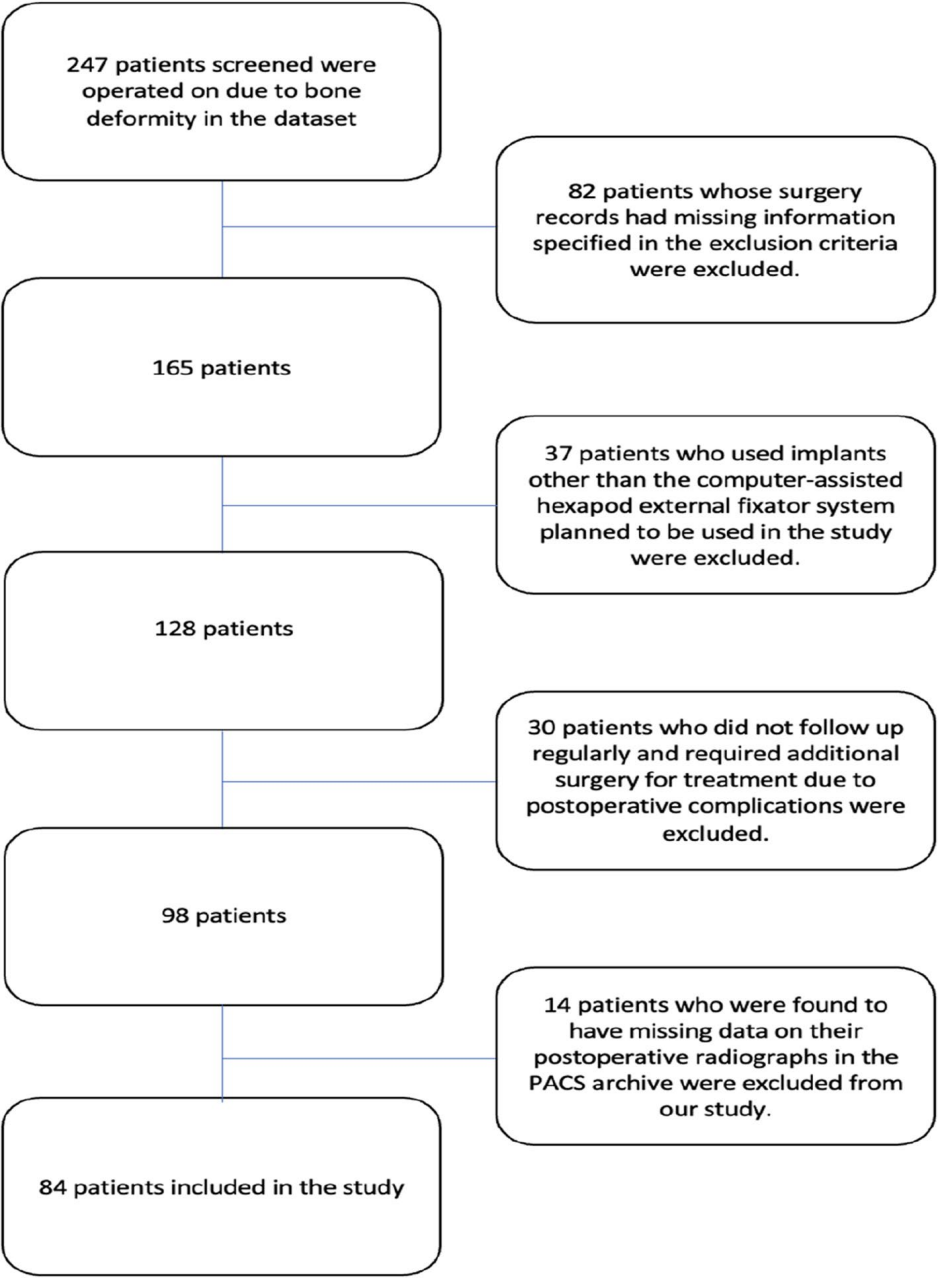
Study flow chart of screened, excluded and analyzed patients

Our study was designed retrospectively, adhered to the Helsinki Declaration, and was approved by the institutional ethics committee (number 3749). Between 2013 and 2022, a cohort of 247 patients who underwent surgery for bone deformities were selected for study. These patients were operated on by two senior surgeons with extensive experience in deformity surgery during this period. The records of these patients were subsequently reviewed by another surgeon who was not involved in the operations at a single center. One surgeon conducted peroperative measurements, while the other performed postoperative measurements. Only patients treated with Ca-CEF, ranging from 8–70 years old, were included. The exclusion criteria included patients lacking anesthesia and surgery start/finish times, missing company details, use of Ca-CEF from a different manufacturer than planned, absence of fluoroscopy records, unspecified measurement techniques for assembly parameters, loss to follow-up, and the need for additional surgery due to postoperative complications. We excluded 82 patients from the study because of lack of data. An additional 37 patients were excluded because they received implants other than the planned Ca-CEF (Spider Frame-Tasarımmed, Istanbul, Turkey). As a result, patients who were plated and had acute deformity correction were excluded from the study, leaving only the complex deformity patient group treated with Ca-CEF. Moreover, 30 patients were excluded because of loss to follow-up and the need for additional surgical procedures to address postoperative complications. Upon reviewing postoperative radiographs in the PACS archive, 14 patients were excluded because of incomplete data, and 84 patients met the inclusion criteria in our study. The assembly parameters of 37 patients were calculated on the basis of peroperative measurements, and those of 47 patients were calculated on the basis of postoperative measurements (Fig. 1). For all patients, we recorded the total AP and lateral plane deformity, surgical duration, peroperative fluoroscopy dose, number of radiographs taken within the first day, first week, and first month postoperatively, and total number of radiographs taken before deformity correction.

### Technique

**Fig. 2:**
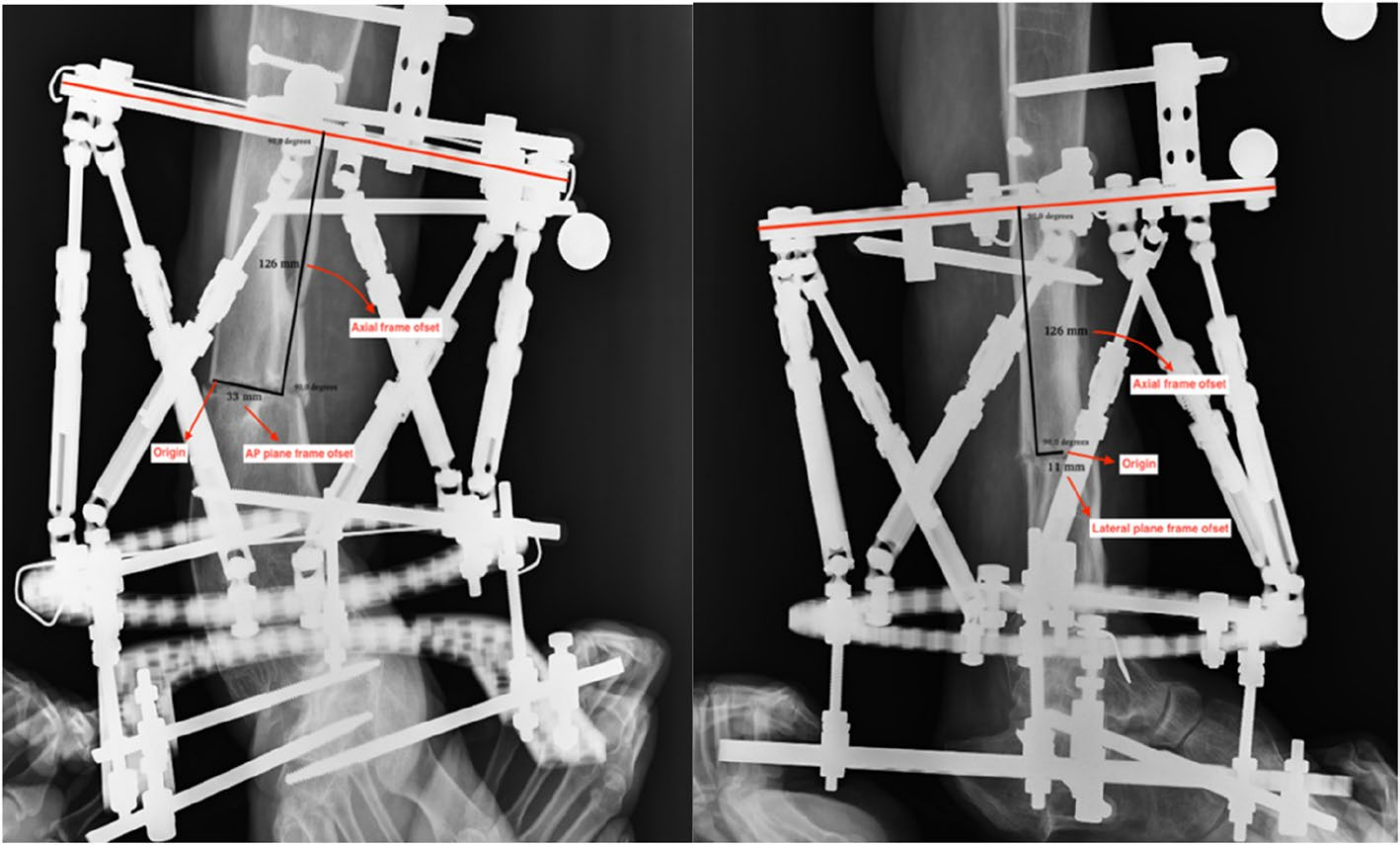
Mounting parameters shown on proximal reference radiograph of a tibial deformity

To achieve proper correction of deformities and fractures and to create correction schedules for patients, it is essential to accurately measure and input frame, assembly, and deformity parameters into the system's software application. The frame parameters include the dimensions of the rings applied to the patient, the lengths of the telescopic rods, and their initial lengths upon system setup. Deformity parameters encompass angular deformities in the AP and lateral planes, the amount of translation and direction, the amount of shortening or lengthening, and the degree and direction of rotation. In addition to rotation, deformity parameters can typically be measured via radiography and fluoroscopy, whereas rotation may require clinical examination or CT. Assembly parameters involve measurements relative to the frame, referencing the location of the virtual hinge termed the starting point relative to the reference ring that facilitates bone positioning, the osteotomy line, and the point of deformity correction, typically at the apex but variable as per surgeon preference. When measuring assembly parameters regardless of the method, the distance from the apex of the deformity to the reference ring can lead to projection errors, resulting in inaccurate measurements of the parameters. To prevent this, the reference ring should be positioned as close to the apex of the deformity as possible. These measurements include four key aspects: anterior–posterior frame offset, lateral frame offset, axial frame offset, and rotational offset of the reference ring. For radiological determination of the assembly parameters, the X-ray must be perpendicular to the plane of the reference ring to ensure that it appears as a straight line in the AP and lateral radiographs. The anterior–posterior frame offset is the perpendicular distance between the midpoint of the reference ring in AP radiographs and the origin. The lateral frame offset is the perpendicular distance between the midpoint of the reference ring in lateral radiographs and the origin. The axial frame offset refers to the distance between the reference ring and the origin. The rotational frame offset indicates the rotation relative to the reference bone (Fig. 2).

**Fig. 3:**
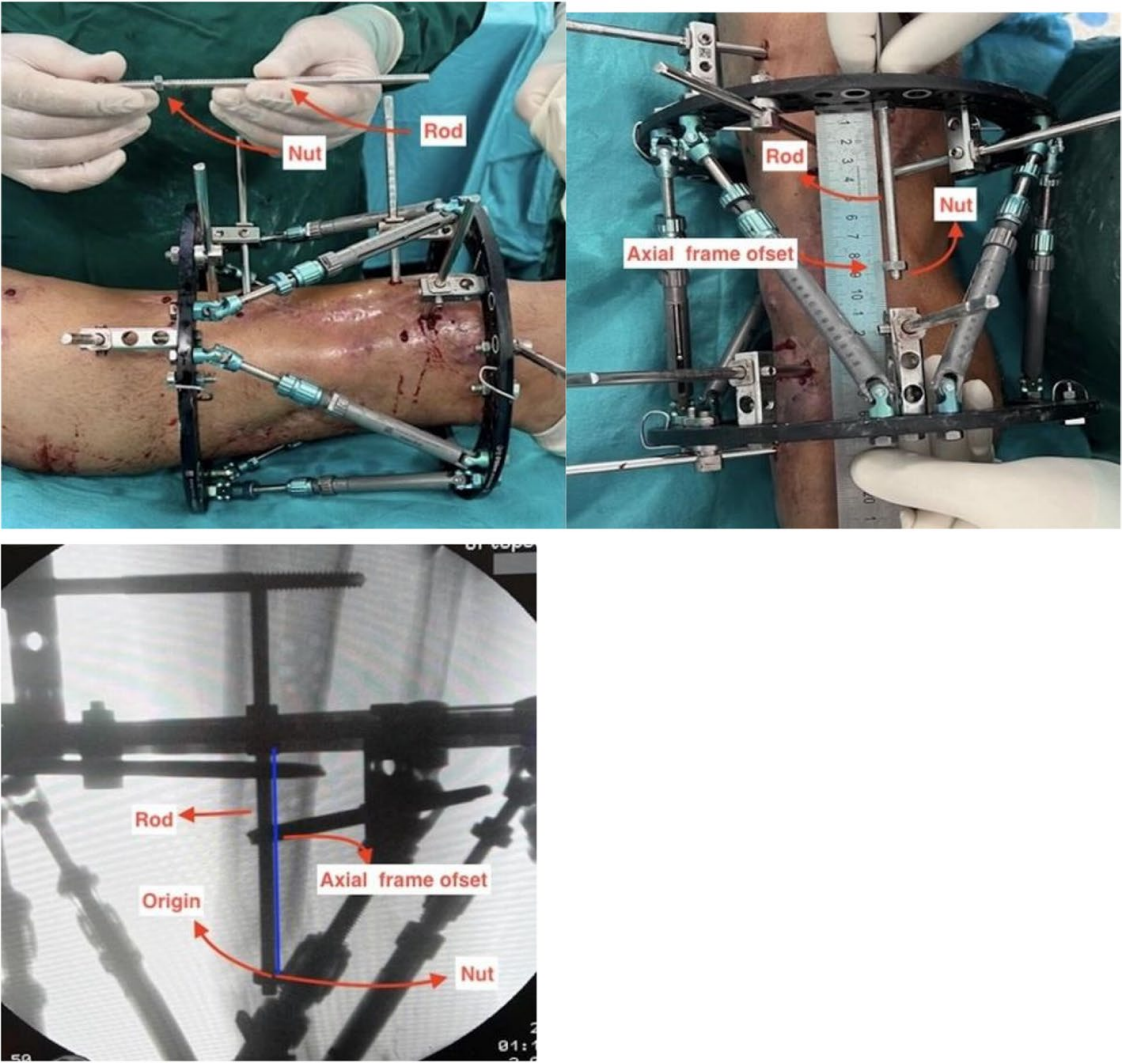
The threaded rod and nut were placed at the midpoint of the proximal ring in the AP plane, and the axial frame offset was measured by obtaining a vertical image of the fluoroscopic ring

Spider frame (Tasarımmed, Istanbul, Turkey) was used as the Ca-CEF, and Spiderfix software (The Spiderfix artificial neural network software Tasarımmed, Istanbul, Turkey) was used for deformity correction. A similar method was described by Gantsoudes et al. [8]. was used. A connecting rod was placed in the proximal ring anterior‒posterior plane, perpendicular to the midpoint of the anterior side of the ring, and fixed with one nut each on the proximal and distal sides. The nut on the connecting rod was brought to the origin point on fluoroscopy taken perpendicular to the proximal ring. The axial frame offset was determined by measuring the distance from the nut to the proximal ring with a ruler (Fig. 3).

**Fig. 4:**
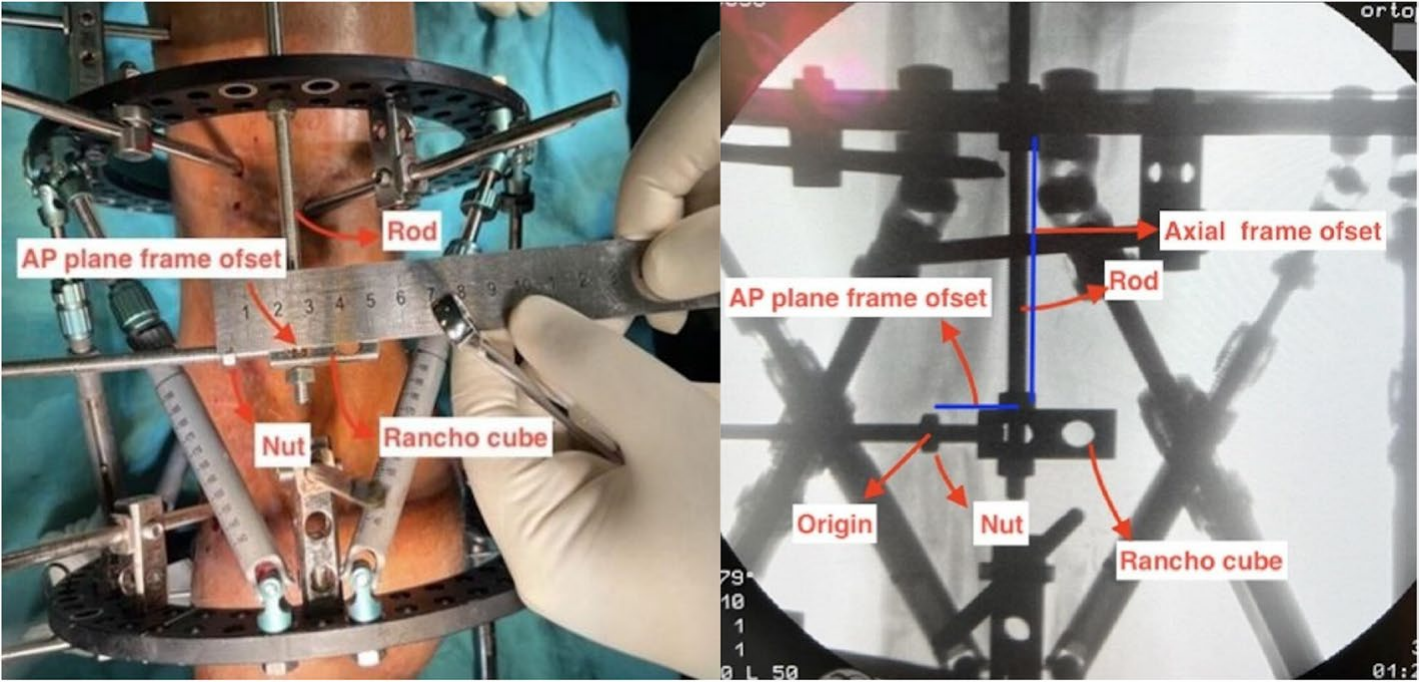
The grooved rod, nut, and rancho cube were placed at the midpoint of the proximal ring in the AP plane, and the anterior posterior frame offset was calculated by obtaining a vertical image of the fluoroscopic ring

A 2-hole Rancho cube and one additional nut were subsequently placed behind the nut. The Rancho cube was positioned such that its projection coincided with the origin. One connecting rod was placed through the Rancho cube parallel to the proximal ring and secured with one nut. The nut was positioned such that its projection on fluoroscopy was aligned with the origin. The distance between the nut, which projected to the origin, and the threaded rod placed perpendicular to the proximal ring was subsequently measured with a ruler to determine the anterior‒posterior frame offset (Fig. 4).

**Fig. 5:**
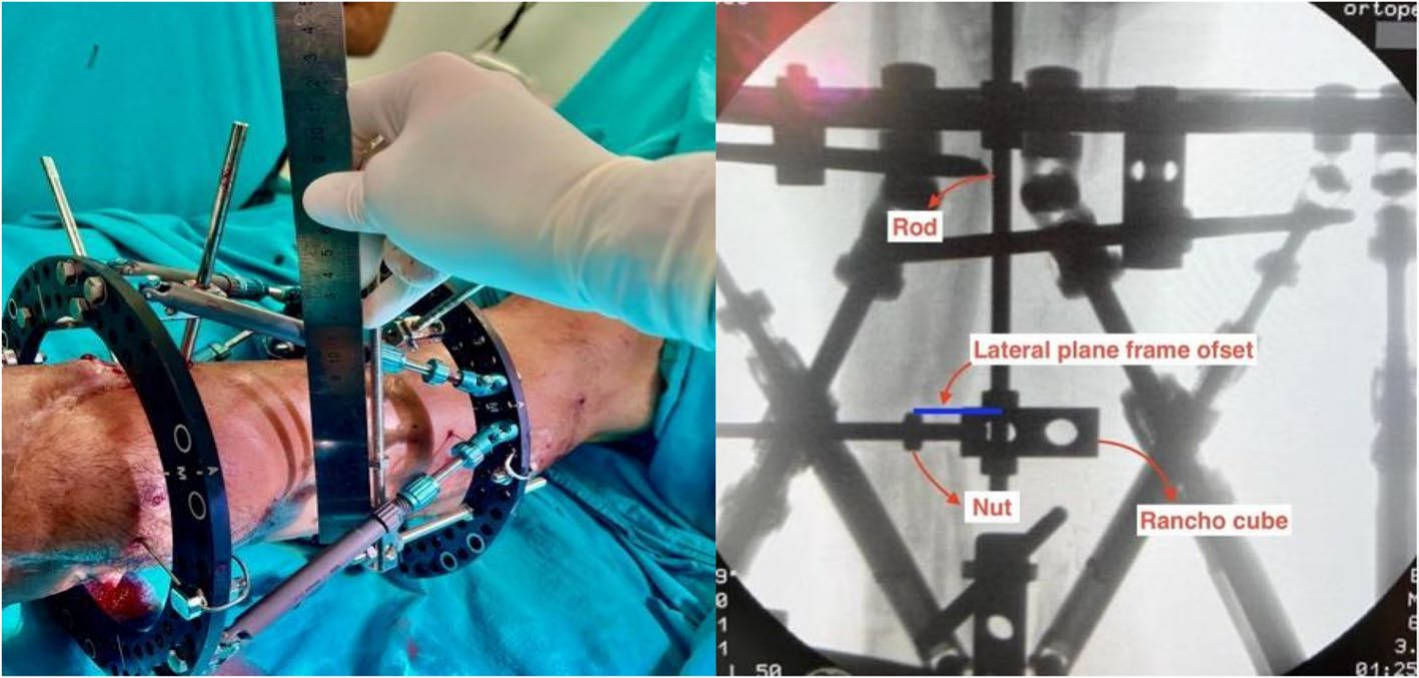
Placing the grooved rod, nut, and rancho cube at the midpoint of the proximal ring in the lateral plane and calculating the lateral frame offset by obtaining a vertical image of the fluoroscopic ring

For the lateral frame offset, a threaded rod was placed perpendicular to the midpoint of the anterior side of the proximal ring in the lateral plane and secured with one nut each on the proximal and distal sides of the ring. A 2-hole Rancho cube and one additional nut were subsequently positioned behind the nut. The Rancho cube was placed between the nuts so that its projection coincided with the origin. One connecting rod is inserted through the Rancho cube, parallel to the proximal ring, and secured with one nut. The nut was positioned such that its projection on fluoroscopy was aligned with the origin. The distance between the nut, which projected the origin, and the threaded rod placed perpendicular to the proximal ring was subsequently measured with a ruler to determine the lateral frame offset (Fig. 5).

All patients had complex deformities, and deformity analysis was performed with AP and lateral radiographs taken on the first postoperative day. Deformity correction was started on the fifth postoperative day in all patients. In patients requiring lengthening, it was done at a rate of 1 mm per day. The daily deformity correction amount was determined by considering the structures at risk.

## Results

A total of 84 patients with bone deformities (*n* = 24 females, *n* = 60 males) between 2013 and 2022 were retrospectively reviewed. The average age was 29 years (range 8–70). All patients had bone deformities and were treated with Ca-CEF. The deformities of 37 patients were corrected with Spiderfix via peroperative fluoroscopy, and postoperative radiographs were obtained for 47 patients. The mean follow-up period was 140 days, during which all deformities were corrected. Total A-P and lateral plane deformity angle degree, surgical times for all patients, peroperative fluoroscopy measurements, and the number of radiographs taken on postoperative day 1, week, and month until deformity correction were recorded (Table 1).

**Table 1:** Analysis of the data

	Measurement	N	Mean	SD	*p*
AP + lateral plane deformity angle (degree)	Postoperative measurement	47	34,95	13,32	0,698
	Perioperative measurement	37	33,45	10,48	
Surgery time (minutes)	Postoperative measurement	47	130,70	56,86	0,045*
	Perioperative measurement	37	155,88	57,20	
Deformity correction time (days)	Postoperative measurement	47	50,24	22,72	0,102
	Perioperative measurement	37	42,31	57,20	
Number of postoperative first day X-ray	Postoperative measurement	47	10,23	1,98	< 0,001*
	Perioperative measurement	37	3,00	0,82	
Number of postoperative first week X-ray	Postoperative measurement	47	14,04	2,89	< 0,001*
	Perioperative measurement	37	5,22	2,09	
Number of postoperative first month X-ray	Postoperative measurement	47	19,49	3,53	< 0,001*
	Perioperative measurement	37	9,41	2,17	
Number of postoperative total X-ray	Postoperative measurement	47	56,04	17,93	< 0,001*
	Perioperative measurement	37	28,70	6,67	
Floroscopi dose (mGy)	Postoperative measurement	47	18,54	13,25	0,105
	Perioperative measurement	37	22,22	14,77	

**Fig. 6:**
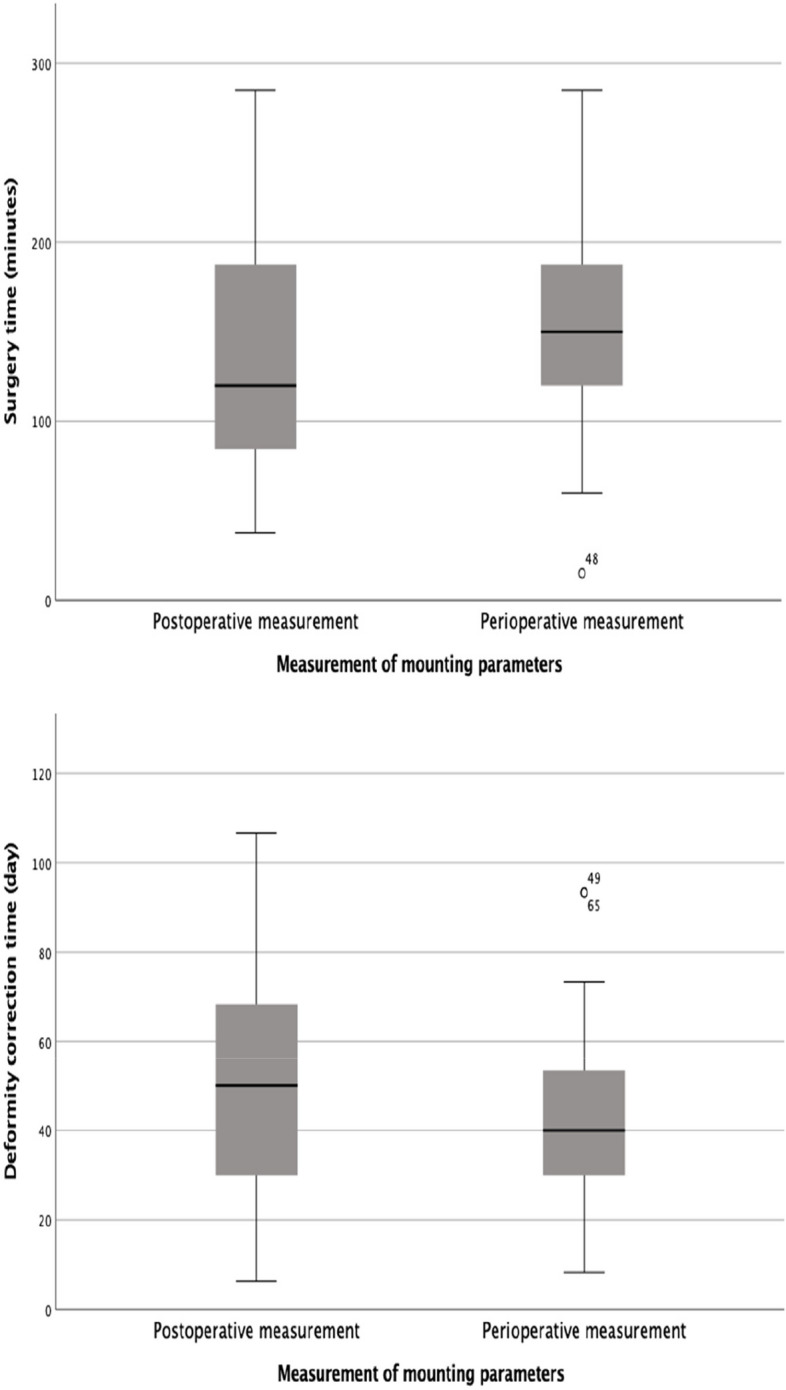
Boxplot of postoperative and peroperative measurements of operative time and deformity correction time

No statistically significant difference was found between preoperative and postoperative measurements in AP + lateral plane deformity angle (degree) measurements (*p* = 0,698). The time to deformity correction was shorter in patients who underwent peroperative measurements (mean 42.31 days, SD 57.2) than in those who underwent postoperative measurements (mean 50.24 days, SD 22.72), although this difference was not statistically significant (*p* = 0.102). However, surgical duration was significantly shorter in patients who had postoperative measurements (mean 130.37 min, SD 56.86) than in those who had peroperative measurements (mean 155.88 min, SD 57.2) (*p* = 0.045) (Fig. 6).

**Fig. 7:**
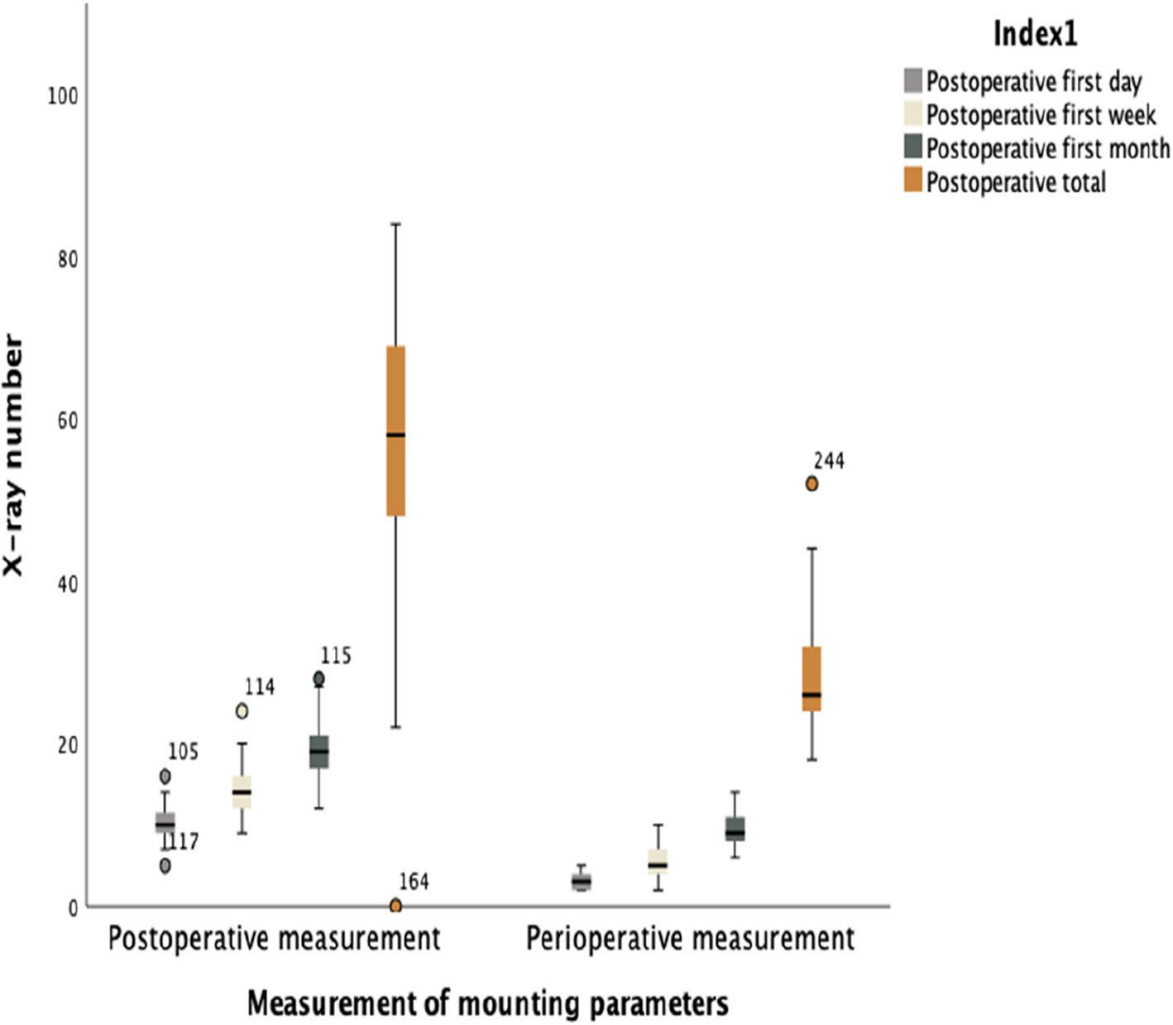
Boxplot of the first postoperative day, week, month, and total number of X-rays

In patients who underwent postoperative measurements, the number of postoperative radiographs taken was 10.23 (SD: 1.98) on the first day, 14.04 (SD: 2.89) at one week, 19.49 (SD: 3.53) at one month, and a total of 56.04 (SD: 17.93). In contrast, patients who had peroperative measurements had fewer postoperative radiographs: 3 (SD: 0.82) on the first day, 5.22 (SD: 2.09) at one week, 9.41 (SD: 2.17) at one month, and a total of 28.7 (SD: 6.67) (*p* < 0.01) (Fig. 7).

**Fig. 8:**
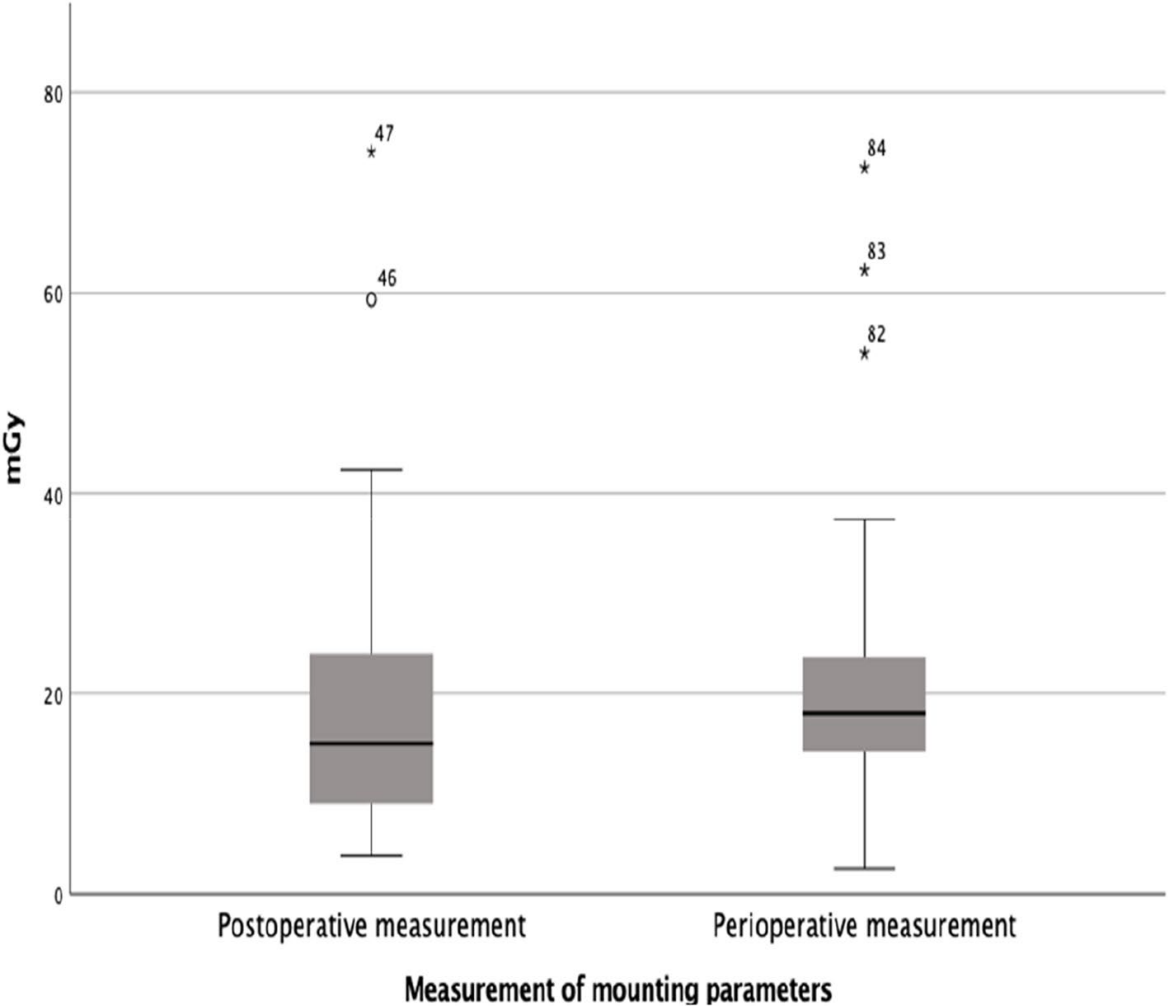
Boxplot of postoperative and peroperative fluoroscopy doses (mGy)

The fluoroscopy dose was 18.54 mGy (SD: 13.25) in patients who underwent postoperative measurements and 22.22 mGy (SD: 14.77) in those with peroperative measurements, with no statistically significant difference (*p* = 0.105) (Fig. 8).

## Discussion

The most important finding of this study is that peroperative fluoroscopy measurements effectively reduce the number of postoperative radiographs, thus reducing radiation exposure for both patients and the surgical team which supports our hypothesis. Furhermore peroperative fluoroscopy increases procedural accuracy and efficiency was supported by the trend toward shorter deformity correction times and decreased postoperative imaging amount, although the increase in surgical time was statistically significant. These findings emphasize that with experienced surgical teams, peroperative fluoroscopy is an effective, safer, and potentially cost-saving alternative to postoperative imaging in deformity correction surgeries.

Ca-CEF theoretically has an accuracy of up to 1/1,000,000 inches and 1/10,000 degrees in correcting deformities. However, achieving perfect placement of this virtual hinge in patients using Ca-CEF requires precise calculation of the deformity, frame, and mounting parameters and accurate input of these parameters into the system [2, 8]. Minimizing exposure to X-rays while accessing mounting parameters and achieving correct parameters can be challenging. Researchers have experimented with various methods to address the difficulty in capturing these specialized radiographs. In a study investigating the average surgical and hospitalization durations, as well as hospital costs associated with the use of Ca-CEF in patients treated for various etiologies, findings reported durations of 277.7 min for surgery, 7.07 days for hospital stay, and $41,507 in hospital costs [11, 12]. These findings underscore the importance of considering the treatment method from a cost perspective.

In previous studies, several methods have been reported for calculating mounting parameters, including postoperative radiography, peroperative fluoroscopy, and computed tomography (CT) [7, 9, 10]. Wright et al. described the silhouette technique for parameter measurement [13]. Deakin et al. aimed to achieve the most accurate measurement via a mounted X-ray guide [14]. Kanellopoulus et al. described a specially designed radiolucent frame that attaches to the TSF (Taylor spatial frame) to guide the acquisition of lateral and anteroposterior radiographs, using a reference ring to achieve perfect orthogonal views in a single exposure, as a noninvasive technique [15]. Liu et al. aimed to improve the accuracy of radiographic measurements by adding a foot frame to the system [16]. Other studies have focused on CT-based measurements and 3D measurement methods to achieve accurate postoperative measurements of mounting parameters [10, 17, 18].

Kucukkaya et al. introduced a postoperative CT measurement technique aimed at preventing errors encountered in radiographic measurement methods [10]. They reported that this method provides a more accurate measurements than radiographic methods do, resulting in lower residual deformity rates. However, they also noted an increased risk of radiation exposure. Gantsoudes et al. described how peroperative measurements can be performed instead of postoperative measurements, highlighting that this method is easier, more repeatable, faster, and cost-effective than other measurement techniques [8]. They utilized a Rancho cube for measuring mounting parameters. Park et al. described a peroperative measurement technique using a calibration ball over fluoroscopy-printed images [19]. Sökücü et al. aimed to determine if there were differences between the mounting parameters measured under fluoroscopy during surgery and those measured on digital radiographs postoperatively [9]. Following their comparison, they reported no significant difference between measurements taken during surgery and those taken postoperatively. They noted that while measurements under fluoroscopy can extend the surgical duration but they provide better visibility than digital radiography does.

On the basis of our peroperative measurement method, similar to the approach described by Gantsoudes et al., we observed that surgeries where peroperative mounting parameters were measured had an average duration of 25.5 min compared with surgeries where postoperative measurements were taken [8]. However, we also noted that the number of radiographs taken postoperatively was significantly lower in terms of the number of peroperative measurements: 70.6% less on the first day, 62.8% less at one week, 51.7% less at one month, and 48.7% less overall. This reduction is attributed to the experienced team's ability to measure mounting parameters accurately peroperatively, which we believe can be achieved more efficiently and with less radiation exposure than measurements performed postoperatively by a less experienced team. Furthermore, the decrease in postoperative radiographs likely reduces patient discomfort during imaging and contributes to cost savings. We also speculate that surgeons feel more confident when they perform peroperative measurements themselves, leading to potentially shorter deformity correction times by an average of 15.7%.

The limitations of this study include its retrospective nature, the small number of patients, the single-center nature of the study, and the fact that the surgeons who performed the operations and used different mounting parameter measurement techniques in the compared groups were not the same person.

## Conclusion

To achieve accurate assembly parameters, minimizing X-ray exposure is crucial but can pose challenges. Our results showed that despite an average increase of 25 min in surgical duration, the time taken for deformity correction was shorter. Additionally, we obtained fewer postoperative radiographs, indicating reduced radiation exposure. In conclusion, our observations suggest that measurements taken in the operating room by an experienced team are conducted more quickly, cost-effectively, and with less radiation exposure.

## Data Availability

No datasets were generated or analysed during the current study.
